# Evaluation of antioxidant and antibacterial properties of dehydrocostus lactone isolated from *Echinops kebericho* root

**DOI:** 10.1002/hsr2.1990

**Published:** 2024-03-21

**Authors:** Sisay Awoke Endalew, Minbale Gashu Taddese, Meseret Muhammed

**Affiliations:** ^1^ Chemistry Department, College of Natural Sciences Wollo University Dessie Ethiopia; ^2^ Chemistry Department, College of Natural and Computational Sciences Debre Berhan University Debre Berhan Ethiopia

**Keywords:** dehydrocostus, DHCL, Echinops, kebericho

## Abstract

**Background and Aim:**

*Echinops kebericho*, an endemic plant to Ethiopia, traditionally used to treat infectious as well as noninfectious diseases. The primary objective of this study was isolating dehydrocostus lactone (DHCL) from *E. kebericho* and evaluating antibacterial activities on selected human pathogenic bacteria.

**Methods:**

Extraction method used in this study was maceration. Based on the bioassay information methanol extract of the root of *E. kebericho* was subjected to column chromatography on silica gel by increasing solvent gradients to isolate DHCL. Optimized amount isolation of DHCL was done by dissolving methanol crude extract by hexane followed by recrystallization at room temperature in the dark place. Different concentrations of the extract were subjected by disc diffusion method against tested bacterial species and antioxidant activity test.

**Results:**

The phytochemical analysis of *E. kebericho* revealed a high presence of terpenoids, which are diverse natural compounds known for their antimicrobial and antioxidant properties. This suggests that terpenoids contribute significantly to the pharmacological effects of *E. kebericho*. In antibacterial testing, *Escherichia coli* was the most sensitive bacterium among all extracts and concentrations. The methanol extract displayed higher antioxidant activity compared to ethyl acetate and hexane extracts, indicating a higher concentration of antioxidant compounds. Notably, the isolated compound DHCL showed promising activity against tested pathogens and significant antioxidant activity. The higher activity of DHCL compared to the crude extracts suggests its responsibility for the observed effects, indicating that the isolation and purification process may have concentrated its beneficial properties. These findings highlight the potential of *E. kebericho* and DHCL as sources of bioactive compounds for therapeutic applications.

**Conclusion:**

All tested extracts and pure compound showed higher inhibition than positive controls in both bioassay. DHCL the principal bioactive component in the root extract of the plant and it displayed potent antibacterial and antioxidant activity.

## 1 INTRODUCTION

Recently, diseases arising from emerging pathogens and sources of free radical initiators become a major treat for medical communities in public health.^1^, ^2^ The spread of antibiotic‐resistant bacteria worldwide becomes a substantial threat to morbidity and mortality.^3^ Mitigation of the current development represents one of the most significant challenges to modern medicine that increased the difficulty of treatment.^1^, ^4^ In this concern, it could be responsible for more than 10 million human deaths per year worldwide by 2050.^5^ That forcing the use of drugs that are more noxious, costlier, and with low efficiency.^6^

Free radicals and oxidants are now a days becomes a global challenge due to the development of chronic and degenerative disease such as cancer, cardiovascular, autoimmune disorders, rheumatoid arthritis, and neurodegenerative diseases.^7^ The counteract mechanism of these oxidative stress can be either naturally generated in situ (endogenous antioxidants), or externally supplied through foods (exogenous antioxidants).^8^ The roles of natural metabolites (antioxidants) are to neutralize the excess of free radicals, to protect the cells against their toxic effects and to contribute to disease prevention.^9^

For primary healthcare more than 80% of people worldwide uses herbal medicine as a means of solution to treat diseases regarding microbial^10^ and accepted as a source for discovering new potent phytochemicals that have been used to treat serious diseases.^11^ Nowadays, the urgent issue in the scientific community is designing a new approaches to deal with drug‐resistant pathogenic bacteria.^12^ Moreover, researchers have been attracting to develop effective of antimicrobial agents by modification of the structure of antibacterial substances.^13^, ^14^ A large number of compounds have been reported as antibacterial and antioxidant agents including essential oils with oxygenated sesquiterpenes.^15^, ^16^, ^17^ In this scenario, researchers are shifting to new alternatives to fight back this concerning situation.^6^ In developing countries preparation of herbal formulations for tackling diseases arising from bacteria as well as oxidants are a long history.^18^

*Echinops kebericho*, endemic to Ethiopia, belongs to a family of Asteraceae.^19^ Traditionally, the smoke of root of this plant uses as the treatment of typhus and fever, moreover, stomach ache can be reduced by chewing the root of Echinops. Ethno‐veterinary medicinal values of root of this plant have been reported as curing intestinal diseases in cattle. Smokes as snake repellent also pronounced in many parts of the country, Ethiopia.^20^ Antileishmanial^21^ and antifungal ^22^ activities of this plant have been reported a potential candidate to treat these diseases and the presence of potentially active ingredients has been reported from the genus Echinops.^23^, ^24^ The medicinal plant species considered in the present study were selected based on its high frequency of mention registered from different ethnobotanical surveys reported in the literature for the treatment of different diseases among endemic plants to Ethiopia. In addition, there is no enough reports of similar studies on pure isolated compound dehydrocostus lactone (DHCL). Therefore, the purpose of this study was isolating DHCL from root of *E. kebericho* to evaluating its antibacterial and antioxidant activities in comparison with different crude extracts.

## 2 MATERIALS AND METHODS

### 2.1 Chemicals and apparatus

Solvents used for extraction are methanol, ethyl acetate, and hexane. Chemical and reagents used for phytochemical investigation were distilled water, sodium hydroxide, ferric chloride, concentrated sulphuric acid, acetic acid, diethyl ether hydrochloric acid, amyl alcohol, chloramphenicol and dimethylsulphoxide (DMSO), and burette reagent. Instruments used during the experiment are thin layer chromatography, test tubes, funnels, beakers, filter paper, hot plates, oven, rotary evaporator attached with vacuum (YC7124), electronic balance (1810‐BA Model), grinder, water bath (Re201BL, Model Indian), petri dish, micropipettes, aluminum foil, measuring cylinder, refrigerator (Samsung model RT34SUMG), incubator (DHP‐9052B Model), separatory funnel, swab, McFarland densitometer (Den‐18 MC model), water distiller, sterilizer. The measurements of NMR‐ spectra were performed with instruments Bruker ACQ 400 AVANCE spectrometer operating at 400 MHz in CDCl_3_. The resonance spectra was recorded using Perkin‐Elmer BX Spectrometer (400–4000 cm^−1^) in KBr. UV spectroscopic data were recorded using T60 UV‐VS spectrophotometer in MeOH.

### 2.2 Plant material collection and extraction

The fresh plant material was bought from Segno Gebeya, Dessie City Administration, about 400 km away from Addis Ababa, during the month of September 2021. The producer reported by Deyno et al.^25^ and Wondiafrash^26^ were applied. The specimen was submitted to Addis Ababa University (AAU) National Herbarium of Ethiopia for identification and deposition voucher number. The sample was authenticated by Melaku Wondafrash and voucher number AAU‐Herbarium‐S1219 for *E. kebericho* was deposited at the National Herbarium of AAU, Ethiopia.

The appropriate research type (design) was comparative experimental design. The dried root (1 kg) was grounded in to coarse powder using electrical mill. One hundred and eighty grams of powdered sample was macerated with 1 L n‐hexane, ethyl acetate and methanol for 72 h for each solvent with continuous shaking. The extracts were filtered through Whatman filter paper (No.1) and concentrated by Rotary evaporator at reduced pressure at 40°C. The resulting semidried extract was then stored in refrigerator below 4°C until used for antimicrobial and antioxidant activities and the preliminary phytochemical analysis.

### 2.3 Phytochemical screening

The preliminary phytochemical analysis of the extracts was carried out using standard procedures to identify the various constituents.

#### 2.3.1 Test for alkaloids

Three milliliters of the extract was added to 5 mL of Hager's reagent (saturated picric acid solution). A yellow precipitate produced immediately an indication of alkaloids.^27^

#### 2.3.2 Test for flavonoids

One milliliter of the extract, a 5 mL of dilute ammonia added and 1 mL conc.H_2_SO_4_. A yellow color was produced, which indicated the presence of flavonoids.^28^

#### 2.3.3 Test for saponins

Three drops of the extract mixed with 5 mL of water. After 15 min form foam indicated the presence of saponins.^28^, ^29^

#### 2.3.4 Test for tannin

Three drops of test solution with gelatin solution white precipitate formed indicate the presence tannin.^30^

#### 2.3.5 Test for carbohydrate

Three drops of test solution with five drops of Benedict's reagent (alkaline solution containing cupric citrate complex) boiled it in water‐bath at 70°C for 10 min and formed a reddish brown precipitate showed the presence of carbohydrate.^31^

#### 2.3.6 Test for steroids

One milliliter of the extracts was dissolved in 10 mL of chloroform and equal volume of concentrated H_2_SO_4_ was added by sides of the test tube. The upper was layer turned to red and the acidic layer showed yellow color with green fluorescence that indicates the presence of steroids.^31^

#### 2.3.7 Test for coumarins

Two milliliters of extract added 3 mL of 10% NaOH yellow color formed. This showed the presence of coumarins.^32^

#### 2.3.8 Test for terpenoids

0.5 mg of the extract was dissolved in 2 mL of chloroform and the addition of 2 mL of Conc.H_2_SO_4_. Then reddish brown color was formed which indicates the presence of terpenoids.^32^

#### 2.3.9 Test for phenols

Three drops of test solution added 5% ferric chloride. Then blue black color formed which indicates the presence of phenols.^33^

#### 2.3.10 Test for anthraquinones

Three drops of test solution added five drops of 2% hydrochloric acid. Appearance of red color indicated the presence of anthraquinones.^29^

### 2.4 Isolation of DHCL from methanol extract

**Figure 1:**
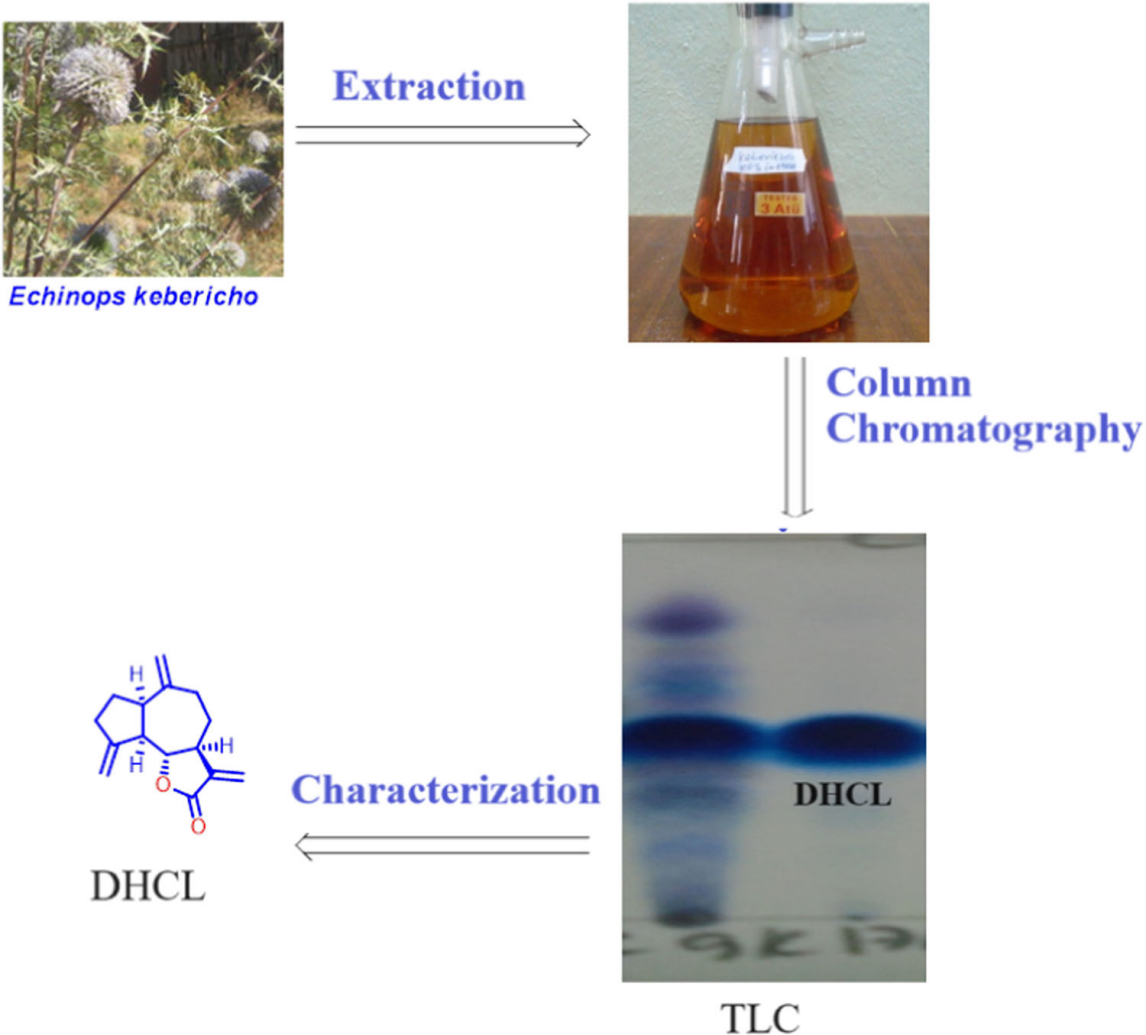
Extraction and isolation method.

Ten grams methanol extract was mixed with silica gel by methanol to adsorb the crude extract for column chromatography. After drying using rotary evaporator brown jelly extract was obtained and then the column was packed with silica gel and hexane. After 1 h, the adsorbed sample was applied to the top of column then the column was eluted using different solvent systems with increasing gradient. By this similar step, many fractions were collected and the TLC of each fraction was checked using different n‐hexane and ethyl acetate solvent ratio. Ten fractions were obtained by mixing fractions with similar spot (similar Rf) and concentrated. Fraction 7 and 9 were observed a promising fraction with white needles crystals at the bottom of the collecting vials. Figure 1 illustrates the extraction and isolation method used in the study. The diagram provides a step‐by‐step visual representation of the process, highlighting the key stages and techniques involved in obtaining the desired compound, DHCL. Finally, these crystals were carefully filtered by Whatman filter paper and a characteristic single violet spots was observed on TLC upon using UV lamp after spraying 1% vanillin sulphuric acid and heating for a few minutes. The resulting purified sample was submitted to spectroscopic analysis.

The amount of DHCL isolated from column chromatography was so small to continue biological assay. So, a large‐scale isolation technique was developed using methanol as an extracting solvent. One thousand grams of powdered root of *E. kebericho* was extracted using 3000 mL of methanol in 5000 mL Erlenmeyer flask for 30 min over hot plate with magnetic stirrer. The crude extract was concentrated using rotary evaporator at reduced pressure to avoid methanol. The resulting crude extract was subjected to solvent‐solvent extraction using n‐hexane and finally the extract was stored at room temperature under dark condition. After 24‐h needle shaped crystals were collected and weighed to give 50 g of pure DHCL.

### 2.5 In vitro antibacterial assay

#### 2.5.1 Test organisms

The bacteria species selected for this study were two gram‐positive namely *Staphylococcus aureus* and *Listeria monocytogenes* and two gram‐negative *Escherichia coli* and *Klebsiella pneumonia*. These microorganisms were obtained from Wollo University Biology Department Laboratory.

### 2.6 Media preparation

Thirty eight milligrams of MHA powder was added into 1 L of distilled water in a flat‐bottomed conical flask. The media was completely dissolved by heating the mixture with frequent agitation until the clear solution was observed. The flask was covered with aluminum foil after tightly closed using cotton wool. The mixture was autoclaved for 15 min at 121°C after which it was left to cool down to room temperature. Seventy milliliters freshly prepared sterile MHA medium was poured into 150 mm diameter Petri dishes. The Petri dishes containing the media were then placed in a sterile and properly adjusted refrigerator until bacteria inoculums were spread on them.^34^

### 2.7 Determination of inhibition zone

Crude extracts (3.5 g) were dissolved in DMSO until the volume of the solution became 1 mL to get 3.5 g/mL stock solutions. Different concentrations of extracts with different solvents (200, 100, 50 mg/mL) were prepared after dilution of the stock solution with DMSO.

Fresh culture bacteria were suspended into 5 mL of sterile normal saline water and then turbidity of suspension was adjusted equivalent to 0.5 McFarland standards by reading on McFarland Densitometer instrument. A sterile cotton swab was dipped into bacterial suspension, rotated gently and pressed firmly on the inside wall of the tube above the fluid level to remove excess inoculums from the cotton swab. The swab was streaked to the entire surface of the MHA plate three times by rotating approximately 60°C each time to ensure an even distribution of the inoculums. Petri‐plates were left for 3 min at room temperature. Sterilized filter papers disk containing different concentrations of plant extract were placed on plate containing MHA.^35^ To measure the turbidity of bacterial suspension spectrophotometer was used. Our absorbance of the suspension measurement was done at the wavelength to 600 nm.

The negative control (5% DMSO in water) and positive control (chloramphenicol, 100 μg/mL) were placed into the labeled agar wells. The plates were placed at room temperature for 2 h and then incubated at 37°C for 24 h. All tests were performed in triplicate for each bacterial species. Finally, the diameters of inhibition zones were measured in millimeter using digital meter. The mean zone of inhibition and standard error of the mean (mean ± SEM) were calculated for the fractions and controls. The zone of inhibition was analyzed whether the bacteria is resistant or sensitive to the reference of a particular antibiotic and values.^36^

### 2.8 Determination of antioxidant activity

Antioxidant activity of different leaves extracts of *E. kebericho* and DHCL were measured by 1, 1‐diphenyl‐2‐picryl hydrazyl (DPPH) following the producer used by Hussen and Endalew.^27^ Briefly, 0.1 mM solution of DPPH was prepared by dissolving 0.004 g of DPPH crystalline solid in 100 mL of analytical grade methanol and stored at 4°C. A 3 mg of the plant extract was dissolved in 10 mL of methanol to prepare 300 μg/mL stock solutions and then serial dilution with methanol was performed to prepare the required concentrated solutions (50, 100, 150, 200, 250, 300 μg/mL) followed the protocol proposed by Hussen and Endalew.^27^ A 2 mL of plant extract solution from each concentration was taken in a test tube and then, 3 mL of DPPH solution was added in each test tube. The same procedure and concentrations were prepared for isolated pure compound, DHCL. After 30 min incubation in the dark, the absorbance at 517 nm was recorded using a UV‐Vis Spectrophotometer.

Reference standard compound being used was ascorbic acid. A stock solution of 800 μg/mL was prepared by dissolving 2 mg ascorbic acid in 2.5 mL of distilled water. Then, serial dilution with different concentrated solution was prepared. Hexane, EtOAc, and MeOH were used as the blank for respective extracts and distilled H_2_O for DHCL. A mixture of 3 mL of 0.1 mM DPPH and (100 μL hexane for hexane extract, 100 μL EtOAc for ethyl acetate extract, 100 μL MeOH for methanol extract and 100 μL H_2_O for DHCL) was used as control. All determinations were performed in triplicate. The percent of inhibition were plotted against concentration from which IC50 values were calculated.

$$\text{DPPH}\;\%\;\text{Inhibition}=\left(A_{\text{control}}-A_{\text{sample}}\right)/A_{\text{control}}\times 100,$$

where, A_control_ is the mixture of methanol/ethanol/water and DPPH solution, and A_sample_ is the mixture of sample extract and DPPH solution.

### 2.9 Statistical analysis

All tests were carried out in triplicate, and data was expressed as mean standard deviation (SD) or standard error of mean (SEM), excel, and Chemdraw (to draw different chemical structure). Differences among means at 5% level (*p* < 0.05) were considered statistically significant.

## 3 RESULTS AND DISCUSSION

### 3.1 Percentage yields and phytochemical screening tests of extracts

The yield of the crude extracts obtained from root of *E. kebericho* using methanol, ethyl acetate and n‐hexane were 19%, 12% and 9%, respectively. The methanol crude extract had the highest percentage yields compared to ethyl acetate and n‐hexane crude extracts. The result of percent yield shows the test plant contains more polar constituents than nonpolar ones. As the phytochemical analysis indicated extracts of *E. kebericho* is rich in various secondary metabolites, such as alkaloids, flavonoids, coumarins, phenols, terpenoids, tannins, and steroids, but there was negative result on carbohydrate and saponins (Table 1).

**Table 1:** Phytochemical screening tests of n‐hexane, ethyl acetate and methanol extracts.

Secondary metabolites	n‐hexane	EtOAc	MeOH
Terpenoids	+	+	++
Flavonoids	+	++	++
Phenols	+	++	++
Saponins	−	−	−
Tannins	+	+	++
Alkaloids	+	++	++
Carbohydrates	−	−	−
Steroids	+	+	+
Coumarins	+	+	+

*Note*: (−) not detected (+) detected.

### 3.2 Characterization of DHCL

**Figure 2:**
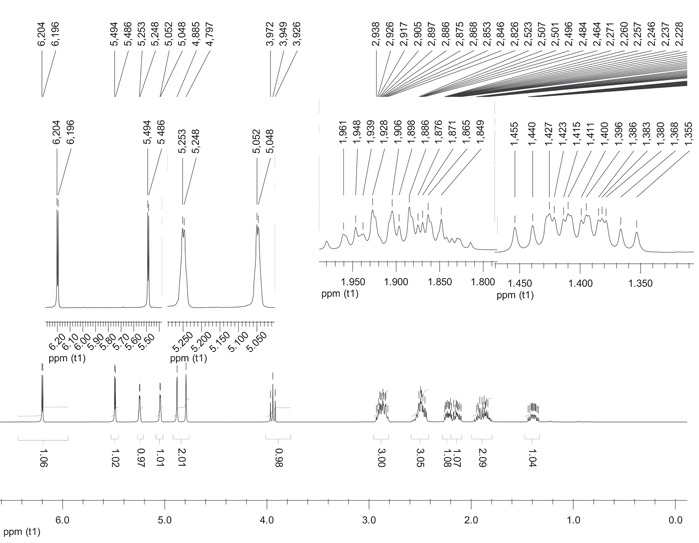
^1^H NMR spectra of dehydrocostus lactone.

**Figure 3:**
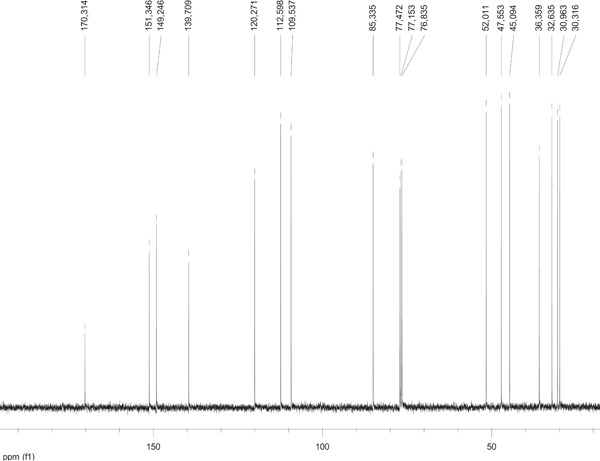
^13^C NMR spectra of dehydrocostus lactone.

DHCL was isolated as white crystals (Rf 0.36 in EtOAc:Hex (1:1); mp 56‐57oC) with a molecular formula C_15_H_18_O_2_ as evidenced by LC‐ESI‐MS which exhibited a molecular ion peak, [M]^+^, at m/z 230.96(100%). The IR spectrum showed characteristic absorptions attributable to conjugated ketone group (1744 cm^−1^) and C = C (1644 cm^−1^). Figure 2 presents the ¹H NMR spectra of DHCL. The peaks in the spectra correspond to different hydrogen atoms in the DHCL molecule, providing valuable information about the molecular structure and chemical environment. The ^1^H NMR spectrum (Table 2) displayed the six exocyclic olefinic protons of which four are observed as doublet because of geminal coupling and were appeared as single. A triplet signals at δ 3.94 (1H, t, J = 9.2) was assigned for proton attached on oxymethine carbon. The remaining multiplet signals at different chemical shifts were assigned for methylene protons on C2, C3, C8, and C9 and Methine protons C1, C5, and C7. Figure 3 displays the ¹³C NMR spectra of DHCL. The peaks in the spectra correspond to different carbon atoms in the DHCL molecule, providing insights into the molecular structure and composition. The ^13^C NMR spectrum (Table 2) of an isolated compound displayed 15 distinct peaks accounted for by 4 methine, 7 methylene and 4 quaternary carbons in the 135 DEPT spectrum. The most downfield signals at δ 170.31 was a characteristics peak of α‐β‐unsaturated lactone functional group. Six olefinic carbons were recorded at a chemical shift of δ 151.35, 149.25, 139.71, 120.27, 112.60, and 109.54 which were assigned for C‐4, C‐10, C‐11, C‐13, C‐14, and C‐15, respectively.

**Table 2:** ^13^C NMR data for DHCL and their literature.

CN	(400 MHz, CDCl_3_)			
	^1^H NMRδ (ppm)	Literature	^13^C NMR	Literature
1	2.91 (1H, m)	2.91 (1H, m)	47.55	47.6
2	1.94 (2H, m) 2.52 (2H, m)	1.94 (2H, m) 2.52 (2H, m)	30.32	30.3 32.6
3	2.52 (2H, m)	2.52 (2H, m)	32.64	32.6 151.3
4	‐	‐	151.35	151.3
5	2.91 (1H, m)	2.91 (1H, m) 3.94 (1H, t, J = 9.2)	45.09	45.1
6	3.94 (1H, t, J = 9.2)	3.94 (1H, t, J = 9.2) 2.91 (1H, m)	85.34	85.3
7	2.91 (1H, *m*)	2.91 (1H, *m*)	52.01	52.0
8	1.42(1H, *m*), 2.20 (1H, *m*)	1.42(1H, *m*), 2.20 (1H, *m*)	30.96	30.9
9	2.20 (1H, *m*), 2.52 (1H, m)	2.20 (1H, *m*), 2.52 (1H, m)	39.36	36.3
10	‐	‐	149.25	149.2
11	‐	‐	139.71	139.7
12	‐	‐	170.31	170.3
13	6.19 (1H, d, J = 3.2) 5.49 (1H, d, J = 3.2)	6.20 (1H, d, J = 3.6), 5.48 (1H, d, J = 3.2)	120.27	120.2
14	4.88 (1H, s), 4.79 (1H, s) 5.24 (1H, d, J = 2.0), 5.04 (1H, d, J = 1.6)	4.88 (1H, *s*), 4.79 (1H, *s*)	112.60	112.6
15	5.25 (1H, d, J = 2.0) 5.04 (1H, d, J = 1.6) 5.05 (1H, d, J = 1.6)	5.24 (1H, d, J = 2.0), 5.04 1H, d, J = 1.6)	109.54	109.5

All olefinic carbons were detected as exocyclic double bonds. The other characteristics signal that appeared at δ 85.34 ppm was an oxymethine carbon of the lactone ring. Thus, on the basis of spectroscopic data and also comparison with the literature data,^37^ structure of the compound was deduced as dehydrocostus lactone (DHCL).

### 3.3 Antibacterial activity

**Figure 4:**
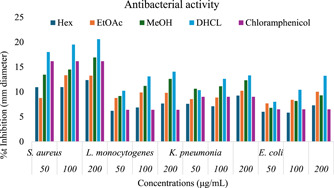
Antibacterial activity of extracts and dehydrocostus lactone.

Antibacterial activity of crude extracts and purely isolated DHCL against selected bacteria was evaluated using disc diffusion methods (Table 3). The extracts and the isolated compound were tested against selected pathogens. Figure 4 showcases the antibacterial activity of the extracts and DHCL and that enables a comparison of the antibacterial efficacy between different extracts and DHCL, providing valuable information on their potential as antibacterial agents. The results showed that the n‐hexane, ethyl acetate, methanol extracts, and DHCL showed antibacterial activities that varied among the bacterial strains. The crude extracts as shown in Table 3 indicated that ethyl acetate and methanol extracts showed better activity than the standard toward all tested organisms. *S. aureus* and *L. monocytogenes* are highly susceptible compared with *K. pneumonia* and *E. coli* because of the nature of the cell‐wall.^38^ It is believed that the inhibitory effects of bioactive compounds are dependent on its ability to damage cell membranes.^39^ Among the tested organisms, *S. aureus* was observed as highly susceptible in a plant extracts.

**Table 3:** Comparison of antibacterial activities (zone inhibition) of crude extracts and isolated compound (DHCL) (mean ± SEM mm diameter).

Bacteria	Conc. (mg/mL)	Types of extract			Compound (μg/mL)	DMSO	Chloramphenicol (100 μg/mL)
		n‐Hexane	Ethyl acetate	MeOH	DHCL		
*Staphylococcus aureus*	50	10.93 ± 0.7	8.77 ± 0.27	13.45 ± 0.12	18.02 ± 0.14	0	16.16 ± 0.3
	100	10.95 ± 0.07	13.32 ± 0.51	14.5 ± 0.89	19.51 ± 0.13		
	200	12.36 ± 0.35	13.26 ± 0.42	16.92 ± 0.21	20.6 ± 0.08		
*Listeria monocytogenes*	50	6.20 ± 0.4	8.78 ± 0.88	9.16 ± 0.74	10.21 ± 0.17	0	6.4 ± 0.4
	100	6.86 ± 0.83	9.88 ± 0.21	11.20 ± 0.2	13.11 ± 0.16		
	200	7.66 ± 0.37	9.79 ± 0.35	12.6 ± 0.2	14.05 ± 0.17		
*Klebsiella pneumonia*	50	7.60 ± 0.2	8.55 ± 0.1	10.61 ± 0.3	10.35 ± 0.4	0	9.02 ± 0.3
	100	7.12 ± 0.7	8.86 ± 0.1	11.11 ± 0.1	12.61 ± 0.2		
	200	9.26 ± 0.3	10.21 ± 0.4	12.32 ± 0.2	13.31 ± 0.4		
*Escherichia coli*	50	6.00 ± 0.2	7.68 ± 0.39	6.8 ± 0.4	8.02 ± 0.30	0	6.5 ± 0.22
	100	5.83 ± 0.21	8.41 ± 0.79	8.17 ± 0.61	10.41 ± 0.11		
	200	7.3 ± 0.12	10.01 ± 0.72	9.3 ± 0.16	13.22 ± 0.14		

The bioassay‐guided fractionation of the methanol extract and further purification of the most antibacterial active fraction led to the isolation and identification of an antibacterial sesquiterpenes lactone, DHCL. As shown in Table 3, this compound exhibited potent antimicrobial activity against tested bacterial species and showed the highest antibacterial activity than both extracts and positive control, chloramphenicol. The data obtained from the present study is comparable with reported data regarding the activity of crude extracts.^25^ The synergic effect of phytochemicals as shown in Table 2 makes all the extracts promising and potent antibacterial agent compared with the standard chloramphenicol. In this study, the pure isolated compound DHCL showed higher activity than all the extracts as well standard.

### 3.4 Antioxidant activity

**Figure 5:**
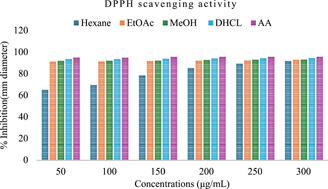
Antioxidant activity of extracts and dehydrocostus lactone (DHCL). Evaluation of antioxidant and antibacterial properties of DHCL Isolated from *Echinops kebericho* Root.

The antioxidant activity of root extracts and DHCL has been studied by its ability to reduce DPPH. Interaction of antioxidant compounds with DPPH is based on the transfer of hydrogen atom or electron to DPPH radical and converts it to 1, 1‐diphenyl‐2‐ picrylhydrazine.^27^ The result of reduction DPPH radicals proved by the transformation of purple color to yellow pale color, which demonstrates the scavenging activity.^40^ The antioxidant activity of the three solvents extracts, DHCL and ascorbic acid against DPPH assay was tested with concentrations ranging from 50 to 300 μg/mL as the results shown in Table 4. Figure 5 demonstrates the antioxidant activity of the extracts and DHCL. The figure allows for a comparison of the antioxidant capacity between different extracts and DHCL, offering insights into their potential as antioxidants.

**Table 4:** Comparison of antioxidant activities of crude extracts and isolated compound (DHCL).

	Hexane extract			EtOAc extract			MeOH extract			DHCL			AA		
Conc. μg/mL	A	%	IC50	A	%	IC50	A	%	IC50	A	%	IC50	A	%	IC50
50	0.812	65.13	109.70	0.165	91.05	95.34	0.127	91.81	94.71	0.115	93.45	93.10	0.089	94.86	91.82
100	0.658	69.37		0.138	91.21		0.112	91.97		0.109	93.61		0.084	94.72	
150	0.532	78.21		0.131	91.56		0.109	92.04		0.106	93.91		0.081	95.44	
200	0.461	85.09		0.123	91.89		0.103	92.57		0.104	94.12		0.076	95.52	
250	0.308	89.11		0.118	92.12		0.100	92.84		0.101	94.34		0.072	95.57	
300	0.254	91.67		0.115	92.84		0.098	93.06		0.099	94.48		0.070	95.68	

As shown in Table 4, both the EtOAc, MeOH and DHCL were displayed a comparable and significant concentration‐dependent free radical scavenging activity from 91.05% to 92.84%, 91.81% to 93.06%, and 93.45% to 94.48%, respectively, compared with that of the standard AA 94.86% to 95.68%. The IC50 values of DPPH assay for the hexane, EtOAc, MeOH, and DHCL were 109.70, 95.34, 94.71, and 93.10, respectively, as compared with standard AA 91.82. Low IC50 values correspond to high antioxidant activity.^41^

Thus, pure isolated DHCL had the highest antioxidant activity compared with crude extracts and EtOH extract showed higher inhibitory activity among the other root extracts. From these results, it was also possible to make several correlations regarding the relationship between the structure of isolated compound DHCL and its DPPH‐scavenging activities. The lactone ring probably enhances the bioactivity of this compound. To the best of our knowledge, the antibacterial activity of dehydrocostus lactone not reported yet.

## 4 CONCLUSION

In this study, phytochemical analysis of n‐hexane, ethyl acetate and methanol extracts of *E. kebericho* roots showed the presence of major secondary metabolites such as terpenoids, flavonoids, tannins, alkaloids, steroids, and coumarins in all extracts. Fractionation of methanol crude extract using n‐hexane over hot‐plate and followed by recrystallization produces DHCL as large‐scale isolation techniques. Antibacterial activity of the extracts as well as pure isolated compound, DHCL, exhibited strong activity against the selected bacterial strains. Among all extracts analyzed in this work, the MeOH extract was the most effective as an antibacterial agent. The antibacterial activity has been attributed to the presence of some active constituents in the extracts. However, the pure compound DHCL showed higher potential than the crude extracts. The antioxidant activity revealed that both extracts and DHCL had comparable activity with a standard AA. The results of an antibacterial and antioxidant activities of this study clearly indicated that *E. kebericho* has the potential to inhibit tested human pathogenic bacteria as well as oxidants in both extract and pure isolate form.

## AUTHOR CONTRIBUTIONS

**Sisay Awoke Endalew**: Conceptualization; supervision; data curation; writing—original draft; formal analysis; methodology. **Minbale Gashu Taddese**: Writing—review & editing. **Meseret Muhammed**: Investigation; methodology; formal analysis; writing—original draft. All authors have read and approved the final version of the manuscript.

## CONFLICT OF INTEREST STATEMENT

The authors declare no conflict of interest.

## TRANSPARENCY STATEMENT

The lead author Sisay Awoke Endalew affirms that this manuscript is an honest, accurate, and transparent account of the study being reported; that no important aspects of the study have been omitted; and that any discrepancies from the study as planned (and, if relevant, registered) have been explained.

## Data Availability

The data sets used and/or analyzed during the current study available from the corresponding author on reasonable request. Sisay Awoke Endalew had full access to all of the data in this study and takes complete responsibility for the integrity of the data and the accuracy of the data analysis.

## References

[1] Luo M , Tang L , Dong Y , Huang H , Deng Z , Sun Y . Antibacterial natural products lobophorin L and M from the marine‐derived Streptomyces sp. Nat Prod Res. 2022;35(24):5581‐5587. 10.1080/14786419.2020.1797730 32713197

[2] Rodrigues LA , Almeida AdC , Gontijo DC , et al. Antibacterial screening of plants from the Brazilian Atlantic Forest led to the identification of active compounds Inmiconia latecrenata (DC.) Naudin. Nat Prod Res. 2022;35(24):5904‐5908. 10.1080/14786419.2020.1802271 32746634

[3] Nocedo‐Mena D , Rivas‐Galindo VM , Navarro P , et al. Antibacterial and cytotoxic activities of new sphingolipids and other constituents isolated from Cissus incisa leaves. Heliyon. 2020;6(8):e04671. 32923710 10.1016/j.heliyon.2020.e04671 PMC7475184

[4] Geers AU , Buijs Y , Strube ML , Gram L , Bentzon‐Tilia M . The natural product biosynthesis potential of the microbiomes of Earth—bioprospecting for novel anti‐microbial agents in the meta‐omics era. Comput Struct Biotechnol J. 2022;20:343‐352. 35035787 10.1016/j.csbj.2021.12.024 PMC8733032

[5] Alonso‐Montemayor FJ , Reyna‐Martínez R , Neira‐Velázquez MG , Sáenz‐Galindo A , Aguilar CN , Narro‐Céspedes RI . A review on antibacterial and therapeutic plasma‐enhanced activities of natural extracts. J King Saud Univ Sci. 2021;33(6):101513.

[6] Anand U , Carpena M , Kowalska‐Góralska M , et al. Safer plant‐based nanoparticles for combating antibiotic resistance in bacteria: a comprehensive review on its potential applications, recent advances, and future perspective. Sci Total Environ. 2022;821:153472. 35093375 10.1016/j.scitotenv.2022.153472

[7] Pham‐Huy LA , He H , Pham‐Huyc C . Free radicals, antioxidants in disease and health. Int J Biomed Sci. 2008;4(2):89‐96. 23675073 PMC3614697

[8] Willcox JK , Ash SL , Catignani GL . Antioxidants and prevention of chronic disease. Crit Rev Food Sci Nutr. 2004;44(4):275‐295. 15462130 10.1080/10408690490468489

[9] Chaudhary P , Janmeda P , Docea AO , et al. Oxidative stress, free radicals and antioxidants: potential crosstalk in the pathophysiology of human diseases. Front Chem. 2023;11:1‐24. 10.3389/fchem.2023.1158198 PMC10206224 37234200

[10] Ekor M . The growing use of herbal medicines: issues relating to adverse reactions and challenges in monitoring safety. Front Pharmacol. 2014;4:177. 24454289 10.3389/fphar.2013.00177 PMC3887317

[11] El‐Saber Batiha G , Alkazmi LM , Wasef LG , Beshbishy AM , Nadwa EH , Rashwan EK . *Syzygium aromaticum* L. (Myrtaceae): traditional uses, bioactive chemical constituents, pharmacological and toxicological activities. Biomolecules. 2020;10(2):202. 32019140 10.3390/biom10020202 PMC7072209

[12] Qin X , Emich J , Goycoolea F . Assessment of the quorum sensing inhibition activity of a non‐toxic chitosan in an N‐acyl homoserine lactone (AHL)‐based *Escherichia coli* biosensor. Biomolecules. 2018;8(3):87. 30181497 10.3390/biom8030087 PMC6164843

[13] El‐Zayat MM , Eraqi MM , Alfaiz FA , Elshaer MM . Antibacterial and antioxidant potential of some Egyptian medicinal plants used in traditional medicine. J King Saud Univ Sci. 2021;33(5):101466.

[14] Zimet P , Valadez R , Raffaelli S , Estevez MB , Pardo H , Alborés S . Biogenic silver nanoparticles conjugated with nisin: improving the antimicrobial and antibiofilm properties of nanomaterials. Chemistry. 2021;3(4):1271‐1285.

[15] da Costa JS , da Cruz ENS , Setzer WN , da Silva JKR , Maia JGS , Figueiredo PLB . Essentials oils from Brazilian eugenia and syzygium species and their biological activities. Biomolecules. 2020;10(8):1155. 32781744 10.3390/biom10081155 PMC7466042

[16] Xavier JKAM , Alves NSF , Setzer WN , da Silva JKR . Chemical diversity and biological activities of essential oils from licaria, nectrandra and ocotea species (Lauraceae) with occurrence in Brazilian biomes. Biomolecules. 2020;10(6):869. 32517106 10.3390/biom10060869 PMC7356694

[17] da Costa JS , Barroso AS , Mourão RHV , da Silva JKR , Maia JGS , Figueiredo PLB . Seasonal and antioxidant evaluation of essential oil from *Eugenia uniflora* L., curzerene‐rich, thermally produced in situ. Biomolecules. 2020;10(2):328. 32092893 10.3390/biom10020328 PMC7072495

[18] Kiyekbayeva L , Mohamed NM , Yerkebulan O , et al. Phytochemical constituents and antioxidant activity of *Echinops albicaulis* . Nat Prod Res. 2018;32(10):1203‐1207. 28475371 10.1080/14786419.2017.1323213

[19] Ayalew H , Tewelde E , Abebe B , Alebachew Y , Tadesse S . Endemic medicinal plants of Ethiopia: ethnomedicinal uses, biological activities and chemical constituents. J Ethnopharmacol. 2022;293:115307. 35452775 10.1016/j.jep.2022.115307

[20] Calixto JB . Efficacy, safety, quality control, marketing and regulatory guidelines for herbal medicines (phytotherapeutic agents). Braz J Med Biol Res. 2000;33(2):179‐189. 10657057 10.1590/s0100-879x2000000200004

[21] Tariku Y , Hymete A , Hailu A , Rohloff J . In vitro evaluation of antileishmanial activity and toxicity of essential oils of *Artemisia absinthium* and Echinops kebericho. Chem Biodiversity. 2011;8(4):614‐623. 10.1002/cbdv.201000331 21480507

[22] Beressa TB , Deyno S , Alele PE . Antifungal activity of the essential oil of echinops kebericho mesfin: an in vitro study. Evid Based Complementary Altern Med. 2020;2020:3101324. 10.1155/2020/3101324 PMC7676924 33273951

[23] Fokialakis N , Cantrell CL , Duke SO , Skaltsounis AL , Wedge DE . Antifungal activity of thiophenes from *Echinops ritro* . J Agricult Food Chem. 2006;54(5):1651‐1655. 10.1021/jf052702j 16506815

[24] Wang Y , Li X , Meng D‐L , Li Z‐L , Zhang P , Xu J . Thiophenes from Echinops latifolius. J Asian Nat Prod Res. 2006;8(7):585‐588. 17135040 10.1080/10286020500176724

[25] Deyno S , Hope D , Bazira J , Makonnen E , Alele PE . Antibacterial activities of essential oil and fractions of ethanolic extract of Echinops kebericho tuber. 2020;11:608672. 10.3389/fphar.2020.608672 PMC7883827 33597879

[26] Wondafrash M . A Preliminary Guide to Plant Collection, Identification and Herbarium Techniques. Addis Ababa The national Herbarium AAU; 2008.

[27] Hussen EM , Endalew SA . In vitro antioxidant and free‐radical scavenging activities of polar leaf extracts of Vernonia amygdalina. BMC Complement Med Ther. 2023;23(1):146. 37143058 10.1186/s12906-023-03923-y PMC10157976

[28] Patle TK , Shrivas K , Kurrey R , Upadhyay S , Jangde R , Chauhan R . Phytochemical screening and determination of phenolics and flavonoids in Dillenia pentagyna using UV‐vis and FTIR spectroscopy. Spectrochim Acta, Part A. 2020;242:118717. 10.1016/j.saa.2020.118717 32745936

[29] Le NM , Cuong DX , Thinh PV , et al. Phytochemical screening and evaluation of antioxidant properties and antimicrobial activity against xanthomonas axonopodis of *Euphorbia tirucalli* extracts in Binh Thuan Province, Vietnam. Molecules. 2021;26(4):941. 33578946 10.3390/molecules26040941 PMC7916649

[30] Hayat J , Akodad M , Moumen A , et al. Phytochemical screening, polyphenols, flavonoids and tannin content, antioxidant activities and FTIR characterization of *Marrubium vulgare* L. from 2 different localities of Northeast of Morocco. Heliyon. 2020;6(11):e05609. 33305038 10.1016/j.heliyon.2020.e05609 PMC7708819

[31] Manuja A , Rathore N , Choudhary S , Kumar B . Phytochemical screening, cytotoxicity and anti‐inflammatory activities of the leaf extracts from *Lawsonia inermis* of Indian origin to explore their potential for medicinal uses. Med Chem. 2021;17(6):576‐586. 32081108 10.2174/1573406416666200221101953

[32] Kikowska M , Kruszka D , Derda M , Hadaś E , Thiem B . Phytochemical screening and acanthamoebic activity of shoots from in vitro cultures and in vivo plants of eryngium alpinum L.—the endangered and protected species. Molecules. 2020;25(6):1416. 32244952 10.3390/molecules25061416 PMC7144402

[33] Fecker R , Buda V , Alexa E , et al. Phytochemical and biological screening of *Oenothera Biennis* L. hydroalcoholic extract. Biomolecules. 2020;10(6):818. 32466573 10.3390/biom10060818 PMC7356052

[34] Dorababu A . Recent update on antibacterial and antifungal activity of quinoline scaffolds. Arch Pharm. 2021;354(3):e2000232. 10.1002/ardp.202000232 33210348

[35] Dubreuil L , Jehl F , Cattoen C , et al. Improvement of a disk diffusion method for antibiotic susceptibility testing of anaerobic bacteria. French recommendations revisited for 2020. Anaerobe. 2020;64:102213. 32615269 10.1016/j.anaerobe.2020.102213

[36] Lynch D , Hill C , Field D , Begley M . Inhibition of Listeria monocytogenes by the *Staphylococcus capitis*—derived bacteriocin capidermicin. Food Microbiol. 2021;94:103661. 33279086 10.1016/j.fm.2020.103661

[37] Kumar A , Agnihotri VK . NMR based profiling of sesquiterpene lactones in *Saussurea lappa* roots collected from different location of Western Himalaya. Nat Prod Res. 2022;36(2):621‐624. 32657146 10.1080/14786419.2020.1789635

[38] Martinez de Tejada G , Sanchez‐Gomez S , Razquin‐Olazaran I , et al. Bacterial cell wall compounds as promising targets of antimicrobial agents I. Antimicrobial peptides and lipopolyamines. Curr Drug Targets. 2012;13(9):1121‐1130. 22664072 10.2174/138945012802002410 PMC3694180

[39] Julian WT , Vasilchenko AV , Shpindyuk DD , Poshvina DV , Vasilchenko AS . Bacterial‐derived plant protection metabolite 2,4‐diacetylphloroglucinol: effects on bacterial cells at inhibitory and subinhibitory concentrations. Biomolecules. 2021;11(1):13. 10.3390/biom11010013 PMC7823703 33375656

[40] Akar Z , Küçük M , Doğan H . A new colorimetric DPPH(•) scavenging activity method with no need for a spectrophotometer applied on synthetic and natural antioxidants and medicinal herbs. J Enzyme Inhib Med Chem. 2017;32(1):640‐647. 28262029 10.1080/14756366.2017.1284068 PMC6009954

[41] Tariq S , Umbreen H , Noreen R , et al. Comparative analysis of antioxidants activity of indigenously produced *Moringa Oleifera* seeds extracts. BioMed Res Int. 2022;2022:4987929. 36325499 10.1155/2022/4987929 PMC9618381

